# Body Mass Index, Gestational Weight Gain, and Obstetric Complications in Moroccan Population

**DOI:** 10.1155/2013/379461

**Published:** 2013-07-07

**Authors:** Latifa Mochhoury, Rachid Razine, Jalal Kasouati, Mariam Kabiri, Amina Barkat

**Affiliations:** ^1^Faculté de Médecine et Pharmacie, Université Mohammed V Souissi, Avenue Belarbi El Alaoui, BP 6203, Rabat, Morocco; ^2^LBRCE, Faculté de Médecine et de Pharmacie de Rabat, Université Mohammed V Souissi, Benslimane 13000, Morocco; ^3^Equipe de Recherché en Santé et Nutrition du Couple Mere Enfant, CRECET, Faculté de Médecine et de Pharmacie de Rabat, Université Mohammed V Souissi, Benslimane 13000, Morocco

## Abstract

*Objectives*. To evaluate the impact of the body mass index (BMI) before pregnancy and the weight gain during pregnancy, on the occurrence of maternal and neonatal morbidity in the Moroccan population, as well as to analyze the quality of the weight gain depending on the BMI. *Methods*. A study was carried out over a period of one year from October 1, 2010 to October 1, 2011, using data collected from a descriptive-transversal study. We recruited nondiabetic women without several HTAs, delivering singletons from 37 completed weeks up to 42 weeks gestation. *Results*. Total of 1408 were analyzed. The risks of moderate hypertension, macrosomia, dystocia, and resort to cesarean section were higher among overweight or obese women, as well as among women whose weight gain was >16 kg. The differences were significant <0.05. *Conclusion*. This study demonstrates that overweight women before pregnancy and weight gain during pregnancy are associated with higher risks of maternal and neonatal complications. These data provide ideas on prevention opportunities.

## 1. Introduction

Nowadays obesity is no longer the prerogative of just the rich and those with substantial incomes in developed countries. New eating habits and sedentary lifestyle are major causes of excess of weight among the Moroccan population regardless of social class, with women are more affected than men. According to figures released by the High Commission for Planning (A Moroccan government institution responsible for statistical analysis, planning, and forecasts) around 33.7% of adult population aged 20 and over are affected by overweight (preobese), while 17.5% are classified to be suffering from severe obesity. Furthermore, it is known that maternal nutritional status before and during pregnancy has a significant influence on the fetal development, the health of the newborn, and its development. Complications related to maternal obesity are classified into two groups: on the one hand, those that affect the mother, and which result in gestational diabetes, preeclampsia, cesarean section, postpartum hemorrhage, and wound infections; on the other hand, complications that affect the fetus, newborn, and child development, which includes macrosomia [1], prematurity, and fetal death in utero. 

Surveys in this field have often been based on body mass index before pregnancy (BMI). The influence of gestational weight gain in reference to the different classes of the BMI of the same parturient is rarely studied. Several suggestions for maternal optimal weight gain have been proposed to lead to an appropriate scheme. The Institute of Medicine (IOM) [2] has published recommended weight gain by BMI preconception. The weight gain in terms of these recommendations should be between 12.5 and 18 kg if the BMI is inferior to 19.8 kg/m^2^. In case the BMI is between 19.8 and 26.0 kg/m^2^, the weight gain should be between 11.5 and 16 kg. In addition, if the BMI is >26.0 to 29.0 kg/m^2^, the weight gain should be between 7.0 and 11.5 kg, and finally a BMI > 29.0 kg/m^2^ evolves a weight gain not exceeding 7.0 kg. A few studies have evaluated patterns of weight gain based on the body mass index in developing countries, mostly among the population of North America and Europe, where anthropometric characteristics are different from the Moroccan population. Thus, the aim of this study is to evaluate the effects of body mass index (BMI) before pregnancy and weight gain during pregnancy and its relationship to the occurrence of maternal and neonatal morbidity among the Moroccan women population as well as to analyze the quality of weight gain depending on BMI.

## 2. Methods

### 2.1. Study Population and Design

A descriptive-transversal quantitative study at the Maternity Department of Hassan II Hospital in Benslimane, a town located in the north-west of Morocco, 60 km far away of the capital Rabat, which has a population of 22,000 inhabitants. The study was carried out over a period of one year from October 1, 2010 to October 1, 2011.

The study included only women who knew their weight before pregnancy and who had prenatal care before 12 WG. The weight measurement during this consultation confirmed the weight before pregnancy reported by women. Were excluded abortions before 22 weeks of gestation (WG) and fetal deaths to prevent such accidents from other causes than maternal weight; twins, mothers with more hypertension, ignoring the date of their last menstrual period or missing weight, diabetic women to avoid this pathology is a confounding factor of macrosomia; it's directly related to birth weight.

Information on the determinants and covariates was obtained from a questionnaire. Maternal prepregnancy body mass index (BMI) was categorised in four classes: underweight: <18.5, normal weight: 18.5–24.9, overweight: 25–29.9, and obesity: >30 kg/m^2^. Gestational weight gain was defined as the difference between the maternal weight at birth and the maternal weight recorded at the first visit to the hospital. Gestational weight gains were grouped into three categories as low weight gain (<8.0 kg), normal weight gain (8 to 16.0 kg), and high weight gain (over 16 kg). 

Trained female investigators administered questionnaires every day including weekend and inquiring *women* about the following: age, marital status, income, years of education, marital status, number of previous births, date of birth, and date of last menstrual period.

The questionnaires were completed by accessing prenatal care and medical records during the period of hospitalization for delivery.

#### 2.1.1. Data Analysis

The parameters collected in survey forms were stored, coded, and analyzed using Statistical Package for Social Sciences (SPSS) 13.0 (SPSS, Inc., Chicago, IL, USA). Quantitative and qualitative variables were created from the data, which were codified for the statistical analysis. The descriptive analysis of the variables was based primarily on class size and proportions, and mean and standard deviations were used as measures' of central tendency and dispersion. 

Regarding the conditions, qualitative variables were compared using the chi-square test or Fisher's exact test. Pearson correlation test was performed to understand the relationships between quantitative variables. Means comparison of quantitative variables for different classes of a qualitative variable were performed using the Student's *t*-test for independent samples, after verification of the different condition of the test. For all statistical tests, a *P* threshold of <0.05 was considered significant.

#### 2.1.2. Operational Definitions

In the survey, we considered as urban all women living in the town and as rural those living 10 km or further from the city Centre.

*Monthly Income.* According to a study made in 2007 by the High Commission for Planning (HPC) a net monthly income of less than 3,000 Moroccan Dirhams (MAD) was classified as low, whereas a monthly net income of 3,000 MAD or higher this was classified as middle income. 

Fundal height is represented by the distance between the symphysis pubis and the fundus of the uterus.

Newborns' weight was determined in the immediate aftermath of childbirth using Seca medical scales.

The outcome of interest was macrosomia, defined as a birth weight of at least 4000 grams (g); low birth weight is below 2,600 g as in previous studies [3].

Shoulder dystocia occurs when the baby's shoulders get stuck during delivery of the baby.

Gestational hypertension occurs when the systolic blood pressure is ≥140 mm Hg or diastolic blood pressure ≥90 mm Hg, measured at two different intervals with woman at rest for more than 15 minutes.

A postpartum hemorrhage is defined as blood loss superior to 500 mL in the first 24 hours after delivery.

Infections are endometritis and infections of the genitourinary tract.

## 3. Results

We included in our survey 1,408 parturients admitted for delivery.  Table 1 shows the main characteristics of the sample population. Parturients were divided into four groups according to their BMI before pregnancy (BMI) = *P* (kg)/*T* (m²). It was considered as underweight any parturient with a BMI < 20 kg/m² (*N* = 102), as normal weight those with a BMI between 20 kg/m² and 24.9 kg/m² (*N* = 871), those with a BMI between 25 kg/m² and 29.9 kg/m² were classified as overweight (*N* = 348), whereas a BMI ≥ 30 kg/m² we refers obesity (*N* = 87). Furthermore, we classified women into three groups depending on the weight gain during pregnancy.

 Group I: weight gain <8 kg (*N* = 530).
 Group II: weight gain between 8 and 16 kg (*N* = 777).
 Group III: weight gain >16 kg (*N* = 101).

### 3.1. Effects of Prepregnancy Weight and Pregnancy Weight Gain

Among the 1,408 women investigated, the rate of obese parturient was much smaller and younger. The average ages and sizes were, respectively, (30 years ± 6) and (159 ± 8.9 cm); the statistical significance *P* < 0.05. There was also a significant difference depending on the place of residence percentage of obese multiparous women (81.4%) and macrosomia.

Groups in weight gain <8 kg (I and II) were from rural areas more than those of weight gain >16 kg (III), (41, 3%), (51, 8%), and (6, 9%), respectively, with a significant statistical difference. Women whose weight gain was >8 kg (II and III) were taller than those of group I with weight gain <8 kg, with an average size (165.1 ± 5.2 cm) for group III, (161.9 ± 5.6 cm) for group II, and (160.6 ± 5.9 cm) for the group I; with *P* < 0.001. The occurrence of macrocosmic newborn is more likely in women in groups II and III, unlike women in the group I who are susceptible to have fetal low birth weight; statistical difference is significant.

Table 2 shows maternal complications according to BMI and weight gain. According to the prepregnancy BMI, obese women are more vulnerable to hypertension (93.3%); the difference is statistically significant *P* < 0.05. The same applies to postpartum hemorrhage with a number of 19.5% against 12.1%, 11.1%, and 5.6%, respectively, for groups underweight, normal and overweight. The resort to caesarean section is more important in the case of obesity (26.4% against 7.8%, 10.8%, 12.4% for groups underweight, normal, and overweight, respectively, with a significant difference. The frequency of perineal tears was 23%, against 6.9%, 14.8%, and 12.4% for underweight, normal, and overweight, respectively, the difference is significant. Infections tend to be higher in case of obesity (31%) against 6.9%, 14.5%, and 15.5%, respectively, for groups underweight, normal, and overweight, with significant difference. Besides, obese women tend to have more dystocic deliveries, accounting for 31% against 7.8%, 13.7%, and 15.8%, respectively, for underweight, normal, and overweight groups, with significant difference. 

According to weight gain, high blood pressure is proportional to the weight gain. The incidence is more important with 78.6% in women with weight gain over 16 kg with a significant difference. The caesarean section was most common in women whose weight gain above 16 kg (21.8%) with a significant difference. Besides, the occurrence of hemorrhage was shown to be more common in the sample group with weight gain >16 kg (22.8%), and the difference is significant. It is the same for most perineal wounds which affect the group whose weight gain is above 16 kg (36.7%); the difference is significant. Moreover, infections tend to be higher in the group with weight gain above 16 kg affecting 34.7% of cases; the difference is significant. Incidence of dystocia is also high with 43.7%, proportional to the weight gain in group III; the difference is significant.

Table 3 shows neonatal complications according to the BMI and weight gain. According to the BMI before pregnancy, the average newborn weight was 3,186 ± 603 g 3,325 ± 556 g, 3,458 ± 542 g, and 3,705 ± 692 g for underweight, normal, overweight, and obese groups, respectively. Macrosomia prevalence is higher among obese women accounting for 40.2% against 8.8%, 13.7%, and 17% of the same groups, respectively; the difference is significant. Whereas the prevalence of low birth weight is low in obese women with just 2.3% against 6.9%, 3.8 and 3.2% for underweight, normal, and overweight groups, respectively, the difference is not significant. In reference to weight gain, the average newborn weight was 3,782.9 ± 595 g in group III, 3,435 ± 540.4 g for group II, and 3,201 ± 566.7 g for group I. The difference is significant between different groups. The prevalence of low birth weight was higher in group I with 6.2% against 2.5% and 0.9% for groups II and III, and the difference was significant. Macrosomia, on other hand, tends to be higher among women whose weight gain is above 8 kg category, accounting for 45.5% for group III compared to 17.2% for group II and 8% for group I; the difference is significant.

## 4. Discussion

Obesity and excessive weight gain during pregnancy increase the risk of obstetric and neonatal complications, particularly hypertension, caesarean section, and macrosomia. In fact the rate of hypertension was very significant in overweight women. Perlow and Morgan [4] as well as Edwards et al. [5] observed hypertension in pregnancy to be very significantly frequent in obese women. Many other findings have also confirmed the link between hypertension and excessive weight gain. [6, 7]. However, it is difficult to determine whether the weight gain induced the occurrence of vascular complications, or conversely, weight gain is the result of fluid retention frequently present in cases of preeclampsia or gestational hypertension. Larsen et al. [8] showed that the frequency of macrosomia increases with a high BMI. Besides, obese woman seems more likely to experience a macrosomic baby than a woman of normal weight. Pregnancy in obese women is associated with a high rate of fetal macrosomia, which tends to be not dependent on gestational diabetes. The risk of macrosomia depends not only on the weight before pregnancy [9] but also on weight gain during pregnancy [10]. The risks of macrosomia were increased almost 2- and 3-folds among women who gained 0.50 kg per week or more during pregnancy, and those whose weekly weight gain was greater than 0.59 kg per week [7].

Supported by several other studies [3, 11], our finding is in line with Edwards et al.'s study [5], in which they compared two groups of parturients with high BMI and showed that macrosomia was significantly more frequent when weight gain exceeds 8 kg during pregnancy. There is also a linear relationship between maternal glycemia and fetal glycemia [12]. This assumes that it is possible that excess weight gain during pregnancy increases fetal weight by increasing glucose in their blood. Two elements, however, contradict this hypothesis. On the one hand, excessive maternal weight gain does not appear to be related to the occurrence of gestational diabetes [10, 13], and it is rather influenced by preexisting obesity or rapid weight gain before or at the early stage of pregnancy [14]. On the other hand, Madsen and Ditzel showed that in patients with diabetes type 1 the rate of fetal macrosomia increases despite glycemic control [15].

Ducarne et al. [16] found out that the average weight of newborns was influenced by the BMI of their mothers, and there were also a greater number of children in these obese patients who were macrosomic. In this study, it was observed that high body mass index combined with high weight gain was a factor risk for hypertension, macrosomia, and low risk of low birth weight. Cnattingius et al. [1] confirmed the idea and stated that overweight protects against low birth weight. Besides, Kabali and Werler [3] proved that the risk of fetal macrosomia was significantly higher for women who were overweight before pregnancy and for those who gained excessive gestational weight. However, the risk was not increased for women of normal weight before pregnancy who gained excessive gestational weight or for those who were overweight before pregnancy but gained a normal or low gestational weight. Therefore, pregestational BMI and gestational weight gain are major factors in determining birth weight. In fact, macrosomia increases the risk of dystocia in obese patients [17]. Ouzounian et al. [18] affirmed that the risk of dystocia is multiplied by a factor of 4 in case of macrosomic children. Besides, Nesbitt et al. [19] stated that the risk of shoulder dystocia is increased by 5% for newborn whose weight is between 4,000 and 4,250 g and by 21% for newborns whose weight is between 4,750 and 5,000 g. 

Obesity increases the risk of caesarean section for overweight pregnant women compared to women of optimal weight. Crane and his colleagues [20] argue that the frequency of cesarean increases with body mass index. Poobalan et al. [21] conducted a meta-analysis cohort, performed from 1996 to 2007, and found that the risk of cesarean delivery was higher among overweight or obese women than women with a normal BMI. During labor, an influence of weight gain on the birth process was found with a significant rate of cesarean section. For Thorsdottir et al., a weight gain of 20 kg increases both the number of instrumental deliveries and caesarean section [6]. Previous studies show that excessive weight gain increases the risk of having a caesarean or instrumental vaginal delivery. Another study by Ratner and Hammer shows that the incidence of cesarean section is greater when women add 12 kg; the risk increases by a factor of 1.9 [22]. For Deruelle [23], an excessive weight gain during pregnancy increases the risk of cesarean section. His study showed that the cesarean section rate was twice high in obese patients with excessive weight gain compared to those whose gain weight considered normal.

Higher incidence of postpartum hemorrhage was reported in patients with excessive weight gain and overweight. The same result was observed by Deruelle et al. [24], who argued that 13.2% of women with excessive weight gain undergo a postpartum haemorrhage against 6.9% in the control group. These data can be explained by the increase in the number of macrosomia among these women and by the pelvic tissue changes associated with excessive weight gain. 

Infections and endometritis are more common among the obese patients. Obese women are also prone to infections of the genitourinary tract [9], and during pregnancy the proteases, *collagenases*, and *elastases* produced by bacteria can degrade the matrix and collagen of fetal membrane cells and lead to membrane rupture. Additionally, obesity is associated with low-grade inflammation and slightly elevated levels of cytokines such as IL-6, IL-8, and TNF-*α* in body fluids and tissues. 

## 5. Conclusion

This study has shown an association between maternal overweight and obesity and adverse pregnancy outcomes, including notably higher caesarean section rates, fetal macrosomia, postpartum haemorrhage, and gestational hypertension. Hence, there is a need to develop guidelines on weight gain to optimize pregnancy and neonatal outcomes.

Through activities related to the acceleration of the reduction of maternal and neonatal mortality in Morocco, prenatal consultation is recommended by the pregnancy care and screening for risk factors, namely, maternal weight, hypertension, and diabetes which are a source of additional expenses that are straining health budgets in poor countries like ours. It is, therefore, important that measures can be implemented for dietary management to minimize the obstetrical risk through consistent weight loss as follows:

- fight against physical inactivity and weight excess;
- ensure the availability of care for parturients;
- a great focus on single women with low level of education and low income considering them as a population at risk that should be targeted by prevention and education;
- sensitize health professionals to collect reliable information and accurate measurements.

## Figures and Tables

**Table 1:** Patient characteristics. Quantitative variables were expressed in average ± standard deviation and qualitative variables in numbers and percentage.

Groups BMI§WG	BMI kg/m²				*P*	Weight gain (kg)			*P*
	<20	20–24.9	25–29.9	≥30		<8	8–16	>16	
	*n* = 102	*n* = 871	*n* = 348	*n* = 87		*n* = 530	*n* = 777	*n* = 101	
Age (y ± sd)	25 ± 5.7	26 ± 6.3	28 ± 6.2	29 ± 6.2	<0.01*	26.75 ± 6.7	27.36 ± 6	28 ± 6	0.1
Residence *n* (%)					<0.01*				<0.003*
Urban	43 (42.3)	323 (37.1)	161 (46.4)	40 (46)		183 (32.3)	34 (60)	43 (7.6)	
Rural	59 (57.8)	548 (62.9)	186 (53.6)	47 (54)		347 (41.3)	435 (51.8)	58 (6.9)	
Monthly income *n* (%)					0.41				0.68
<5000 DH	98 (96)	81 (93.1)	328 (94.3)	84 (96.6)		500 (37.9)	728 (55.1)	93 (7)	
>5000 DH	4 (3.9)	60 (6.9)	20 (5.7)	3 (3.4)		30 (34.5)	49 (56.3)	8 (9.2)	
Multiparity	53 (52)	542 (62.3)	241 (69.5)	70 (81.4)	<0.01*	318 (35.1)	511 (56.4)	77 (8.5)	<0.01*
Fundal height cm ± sd	30 ± 3.5	31 ± 3.5	32 ± 3.5	34 ± 4.6	<0.01*	31 ± 3.3	32.5 ± 3.5	35.4 ± 4.4	<0.01*
Size (cm ± sd)	164 ± 6	161 ± 5.3	160 ± 5.6	159 ± 8.9	<0.01	160 ± 5.9	161.9 ± 5.6	165.1 ± 5.2	<0.01*
Birth weight g ± sd	3186 ± 603	3325 ± 556	3458 ± 542	3705 ± 692	<0.01*	3201 ± 566	3435 ± 540	3728 ± 595	<0.01*
Weight gain ((kg) *n* (%)					0.15				
<8 kg	36 (6.4)	318 (60)	135 (25.5)	43 (8.1)		—	—	—	
8–16 kg	61 (7.9)	495 (63.7)	185 (23.8)	36 (4.6)		—	—	—	
>16 kg	7 (6.9)	58 (57.4)	28 (27.7)	8 (7.9)		—	—	—	

*Significant (*P* < 0.05).

**Table 2:** Maternal complications according to the BMI and weight gain.

Maternal complications	BMI (kg/m^2^)				*P*	Weight gain (kg)			*P*
	<20	20–24.9	25–29.9	≥30		<8 kg	8 kg–16 kg	>16 kg	
	*n* = 102	*n* = 871	*n* = 348	*n* = 87		*n* = 530	*N* = 777	*n* = 101	
HTA *n* (%)	5 (5.6)	47 (15.4)	20 (44.4)	14 (93.3)	<0.01	33 (11.4)	42 (28)	11 (78.6)	<0.01
Dystocia *n* (%)	8 (7.8)	119 (13.7)	55 (15.8)	27 (31)	<0.01	52 (9.8)	122 (15.7)	35 (34.7)	<0.01
Haemorrhage *n* (%)	6 (5.9)	97 (11.1)	42 (12.1)	17 (19.5)	0.03	44 (8.3)	95 (12.2)	23 (22.8)	<0.01
Infections *n* (%)	7 (6.9)	126 (14.5)	54 (15.5)	27 (31)	<0.01	55 (10.4)	124 (16)	35 (34.7)	<0.01
TP^#^ *n* (%)	7 (6.9)	129 (14.8)	45 (12.9)	20 (23)	0.013	50 (10.6)	120 (17.4)	29 (36.7)	<0.01
Mode delivery					<0.01				
Vaginal delivery *n* (%)	94 (92.2)	777 (89.2)	305 (87.6)	64 (73.6)		473 (89.2)	688 (88.5)	79 (78.2)	
Caesarean section *n* (%)	8 (7.8)	94 (10.8)	43 (12.4)	23 (26.4)		57 (10.8)	89 (11.5)	22 (21.8)	

HTA: hypertension.

TP: perineal trauma.

^#^Vaginal delivery; quantitative variables were expressed in average ± standard deviation, and qualitative variables were expressed in numbers and percentages.

Significant *P* value < 0.05.

**Table 3:** Neonatal complications according to BMI and weight gain.

Neonatal complications	BMI groups kg/m^2^				*P*	Weight gain GP kg			*P*
	<20	20–24.9	25–29.9	≥30		<8 kg	8 kg–16 kg	>16 kg	
	*n* = 102	*n* = 871	*n* = 348	*n* = 87		*n* = 530	*n* = 777	*n* = 101	
Birth weight (g ± sd)	3186 ± 603	3325 ± 556	3458 ± 542	3705 ± 692	<0.01*	3201 ± 566.7	3435 ± 540.4	3782 ± 595	<0.01*
Low birth weight *n* (%)	7 (6.9)	33 (3.8)	11 (3.2)	2 (2.3)	0.312	33 (6.2)	19 (2.5)	1 (0.9)	<0.01*
Macrosomia *n* (%)	9 (8.8)	119 (13.7)	59 (17)	35 (40.2)	<0.01*	42 (8)	134 (17.2)	46 (45.5)	0.01*
Stillbirth *n* (%)	5 (4.9)	32 (3.7)	14 (4)	1 (1.1)	0.5	24 (4.5)	27 (3.5)	1 (0.9)	0.2
Size (cm ± sd)	49.57 ± 3.2	50.15 ± 1.5	50.11 ± 2.2	50.15 ± 2.3	0.04*	50 ± 1.9	50 ± 2	50.2 ± 0.8	0.68
Perimeter crania (cm ± sd)	34.60 ± 1.3	34.83 ± 0.7	34.84 ± 0.9	34.87 ± 0.6	0.04*	34.8 ± 0.8	34.8 ± 0.9	34.7 ± 0.5	0.76

*Significant (*P* < 0.05).

Quantitative variables were expressed in average ± standard deviation, and qualitative variables were expressed in numbers and percentages.
